# Cellular Traction Stresses Increase with Increasing Metastatic Potential

**DOI:** 10.1371/journal.pone.0032572

**Published:** 2012-02-28

**Authors:** Casey M. Kraning-Rush, Joseph P. Califano, Cynthia A. Reinhart-King

**Affiliations:** Department of Biomedical Engineering, Cornell University, Ithaca, New York, United States of America; University of Liverpool, United Kingdom

## Abstract

Cancer cells exist in a mechanically and chemically heterogeneous microenvironment which undergoes dynamic changes throughout neoplastic progression. During metastasis, cells from a primary tumor acquire characteristics that enable them to escape from the primary tumor and migrate through the heterogeneous stromal environment to establish secondary tumors. Despite being linked to poor prognosis, there are no direct clinical tests available to diagnose the likelihood of metastasis. Moreover, the physical mechanisms employed by metastatic cancer cells to migrate are poorly understood. Because metastasis of most solid tumors requires cells to exert force to reorganize and navigate through dense stroma, we investigated differences in cellular force generation between metastatic and non-metastatic cells. Using traction force microscopy, we found that in human metastatic breast, prostate and lung cancer cell lines, traction stresses were significantly increased compared to non-metastatic counterparts. This trend was recapitulated in the isogenic MCF10AT series of breast cancer cells. Our data also indicate that increased matrix stiffness and collagen density promote increased traction forces, and that metastatic cells generate higher forces than non-metastatic cells across all matrix properties studied. Additionally, we found that cell spreading for these cell lines has a direct relationship with collagen density, but a biphasic relationship with substrate stiffness, indicating that cell area alone does not dictate the magnitude of traction stress generation. Together, these data suggest that cellular contractile force may play an important role in metastasis, and that the physical properties of the stromal environment may regulate cellular force generation. These findings are critical for understanding the physical mechanisms of metastasis and the role of the extracellular microenvironment in metastatic progression.

## Introduction

While significant advances have been made in the treatment of primary tumors through surgery, chemotherapy and radiation treatment, a mechanism for effectively diagnosing the likelihood of metastasis remains elusive [1]. Metastasis is the leading cause of death among cancer patients, resulting in over 90% of cancer-related fatalities [2]. Moreover, there is currently no procedure or test that can definitively determine the metastatic potential of a specific tumor. Clinical oncologists routinely rely on pathology reports and historical statistics to determine patient prognosis and to design a course of palliative therapy [3].

Because metastasis has become the primary obstacle in cancer treatment, there is a substantial body of work attempting to discover a biological marker (or set of markers) for metastasis, but with marginal success [4]. Previous studies have linked overexpression of VEGF-D [5], urokinase plasminogen activator [6], the growth factor receptor CXCR2 [7] and activator protein-1 [8] to increased metastatic breast cancer invasion *in vitro* and *in vivo*. Additionally, studies have shown that a combination of genes can affect organ-specific tropism [9]. However, these discoveries have generally not been applicable to multiple cancer types, or even within subtypes of a single cancer. Recently, advances in genetic profiling have led to the identification of a 17-gene expression signature for metastasis in primary adenocarcinomas [10], as well as a 70-gene expression signature for predicting the clinical prognosis of breast cancer [11]. While patients whose tumors contain these expression patterns will benefit from this kind of genetic analysis, it may not be applicable to a broad spectrum of patients with heterogeneous tumor populations. Additionally, while these signatures may show significant statistical correlation with poor prognosis, they are not descriptive of the physical behaviors of the tumor cells that lead to these clinical results.

Alterations in gene expression patterns result in phenotypic changes at the cellular level during cancer progression. As such, the biophysical characteristics of tumor cells may be a more appropriate and accessible clinical indicator than individual genetic alterations. During metastatic invasion, cancer cells encounter a complex and constantly evolving microenvironment [12] consisting of upregulated extracellular matrix proteins [10], [13], [14], different degrees of extracellular matrix (ECM) crosslinking [13], mechanical heterogeneity [15], [16], varying oxygen levels [17], as well as exposure to shear stress and interstitial pressure [12]. To successfully navigate this dynamic microenvironment, tumor cells must generate force to reorganize the basement membrane, invade into surrounding stroma [18], [19], migrate along ECM fibers [20], [21] and transmigrate through the endothelial cell barrier [7] to enter the circulatory or lympathic system. In addition to enzymatically degrading the ECM with matrix metalloproteinases, metastatic cells can use force to mechanically rearrange their ECM to clear a path for migration [18]. There is evidence from *in vivo* imaging that cells use re-oriented fibers as “train-tracks” to guide their migration away from the primary tumor [21]. Traction forces have previously been shown to mediate normal cell migration [22], adhesion [23], [24], mechanotransduction [25], and ECM remodeling [21], [26], [27]. Notably, these processes are also involved in cancer progression. Paszek et al. have shown a marked difference in the magnitude and organization of traction stresses between cancerous and untransformed mammary epithelial cells, suggesting inherent differences in cell force generation in the cancerous phenotype [15]. However, the effects of metastatic potential on force generation have not yet been thoroughly investigated.

Matrix stiffness has been shown to have a distinct effect on force-mediated cellular behaviors including migration [28], [29], [30], [31], adhesion [25], [32], [33], and ECM remodeling [13], [18]. Because metastasizing cancer cells are exposed to both the increased stiffness of the stroma surrounding most solid tumors, as well as more compliant adipose tissue, it is important to understand the effects of a dynamic mechanical environment on cancer cell force generation. Similarly, ligand density has also been shown to have a significant effect on the force generation of non-cancerous cell types, such as endothelial cells and fibroblasts [34], [35], [36]. During cancer progression, the chemical nature of the extracellular matrix experiences significant changes, affecting the number and nature of binding sites available for tumor cell adhesion and migration. Collagen metabolism has been shown to be dysregulated, with elevated expression, increased deposition, and an increase in collagen crosslinking that contributes to the overall stiffening of the surrounding microenvironment [13]. These factors lead to an increase in mammographic density, which has been specifically correlated to an increased risk for the development of breast cancer [37]. An increase in collagen expression has also been clinically linked to metastatic tumors by genetic analysis of tumor biopsies [10]. Therefore, understanding the independent and interdependent relationships between substrate mechanics, collagen density, and force generation is critical for understanding the mechanism(s) driving metastatic progression.

In this study, we investigate traction force generation as a biophysical marker of metastatic potential. We quantify contractile forces of highly metastatic breast, prostate, and lung cancer cell lines compared to non-tumorigenic epithelial cell lines seeded on substrates of varying stiffness and collagen density using traction force microscopy. Here, we show that highly metastatic cancer cells exert significantly increased forces across all matrix properties studied. Moreover, our data show that increased matrix stiffness and collagen density both promote increased traction forces. These findings provide the first evidence to our knowledge that differential force profiles of metastatic cells may aid in determining metastatic potential.

## Methods

### Cell culture

MCF10A mammary epithelial cells (American Type Culture Collection (ATCC), Rockville, MD) were maintained in Dulbecco's Modified Eagle's Media supplemented with 5% horse serum, 20 ng/mL EGF (Invitrogen, Carlsbad, CA), 10 µg/mL insulin, 0.5 µg/mL hydrocortisone, 100 ng/mL cholera toxin (Sigma-Aldrich, St. Louis, MO), and 1% penicillin-streptomycin (Invitrogen) [38]. MDAMB231 highly metastatic breast adenocarcinoma cells (ATCC) were maintained in Minimum Essential Medium supplemented with 10% fetal bovine serum, and 1% penicillin-streptomycin (Invitrogen). PC3 highly metastatic prostate adenocarcinoma cells (ATCC) were maintained in Kaighn's Modification of Ham's F-12 Medium (ATCC) supplemented with 10% fetal bovine serum and 1% penicillin-streptomycin (Invitrogen). PrEC primary human prostate epithelial cells (Lonza, Walkersville, MD) were maintained in PrEGM prostate epithelial cell growth medium (Lonza) supplemented with SingleQuots (Lonza) according to the manufacturer's recommended protocol. BEAS2B bronchial epithelial cells (ATCC) were maintained in BEBM bronchial epithelial cell basal medium (Lonza) supplemented with SingleQuots (Lonza) according to ATCC recommended protocol, and 1% penicillin-streptomycin (Invitrogen). A549 metastatic lung carcinoma cells (ATCC) were kindly provided by Paraskevi Giannakakou (Weill Cornell Medical College) and were maintained in RPMI 1640 supplemented with 10% fetal bovine serum, 1% penicillin-streptomycin, and 1 µg/mL puromycin (Invitrogen). MCF10AT1 transformed mammary epithelial cells and MCF10CA1a metastatic mammary epithelial cells were obtained from the Barbara Ann Karmanos Cancer Institute (Detroit, MI) and were maintained in 1∶1 Dulbecco's Modified Eagle's Media/F12 supplemented with 5% horse serum, 20 ng/mL EGF (Invitrogen), 10 µg/mL insulin, 0.5 µg/mL hydrocortisone, 100 ng/mL cholera toxin (Sigma-Aldrich), and 1% penicillin-streptomycin (Invitrogen) [39]. All cells were cultured at 37°C and 5% CO_2_. Live cell imaging was performed in a custom temperature, humidity, and CO_2_- controlled stage of a Zeiss Axio Observer Z1m inverted phase contrast microscope with a Hamamatsu ORCA-ER camera.

### Substrate synthesis and traction force microscopy

Substrates of various Young's moduli (1–10 kPa) were synthesized with varying ratios of 3–7.5% acrylamide (40% w/v solution, Bio-Rad, Hercules, CA) and 0.1–0.35% N,N′-methylene-bis-acrylamide (2% w/v solution, Bio-Rad) as described previously [24], [40]. Substrate surfaces were functionalized using N-6-((acryloyl)amido)hexanoic acid, synthesized in our lab [34], covalently bound to 0.0001–0.1 mg/mL type I rat-tail collagen (Becton Dickinson, Franklin Lakes, NJ). The Young's modulus (E) of the polyacrylamide substrates was verified as previously described [32], [34], [40]. Traction force microscopy (TFM) was also performed as previously described [40], [41], [42]. Briefly, cells were seeded on polyacrylamide substrates (E = 1–10 kPa) embedded with 0.5-µm fluorescent beads and allowed to adhere overnight. Cells were then imaged in phase and bead distributions were imaged in fluorescence before and after removal of the cell with trypsin. Bead displacement within the substrate was tracked with correlation-based optical flow and converted into a strain field. These strain fields were converted into traction stresses using the LIBTRC analysis library developed by Professor Micah Dembo of Boston University, who invented the basic theory that underlies TFM [41]. Images were processed with LIBTRC software to determine the cellular traction vectors, T, the total magnitude of the force, |*F*|, and the projected cell area. |*F*| is an integral of the traction field over the entire area of the cell,
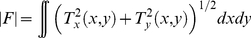
(1)where 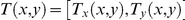 is the continuous field of local traction vectors defined at local spatial coordinates (x,y) in the projected cell area [35]. More than 40 cells were analyzed per condition.

### Statistical tests

Data were compared with analysis of variance (ANOVA) and with post-hoc Tukey's Honestly Significantly Different (HSD) test or Student's *t* test where appropriate in JMP software (v.8, SAS, Cary, NC). All data were logarithmically transformed prior to analysis to ensure normal distribution. All data are reported as mean ± standard error of the mean (SEM).

## Results

### Metastatic cancer cells exert stronger traction forces

To investigate the relationship between cellular traction force generation and metastatic potential, we examined the differences in traction force generation in three independent cancer models: breast, prostate, and lung cancer. These cancers comprise the three most fatal cancers in both men and women [43], with metastatic disease being the ultimate cause of death in over 90% of these patients [2]. During metastatic progression, phenotypic changes in cancer cells result in altered adhesion and migration behavior, allowing cells to escape from the tumor mass into surrounding tissue [12]. Because contractile force is known to mediate these behaviors in normal cells [23], [24], we hypothesized that cancer cells require increased force generation to metastasize. For each type of cancer, we chose a cell line (breast, prostate, lung, respectively) that was previously characterized as highly invasive *in vitro* and metastatic *in vivo* (MDAMB231, PC3, and A549), as well as a non-tumorigenic, non-metastatic control cell population that is representative of healthy native epithelial tissue (MCF10A, PrEC, and BEAS-2B).

**Figure 1:**
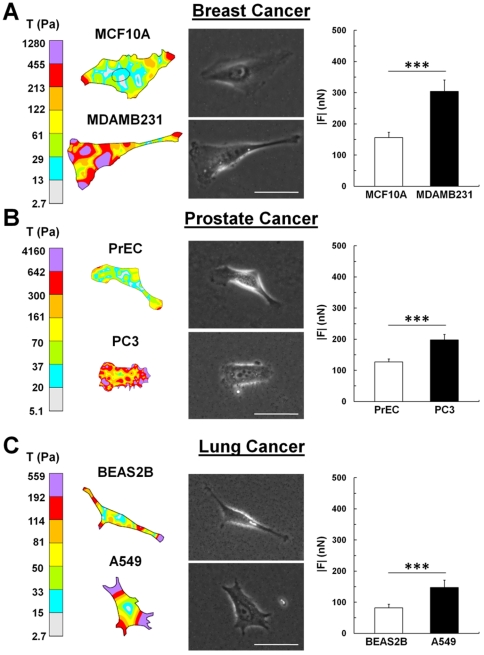
Metastatic cancer cells exert greater forces than non-metastatic cells. (A) Representative traction maps (*left*), corresponding phase images (*middle*), and overall net traction force (|*F*|, *right*) of non-metastatic mammary epithelial (MCF10A) and highly metastatic (MDAMB231) cancer cells. (B) Representative traction maps (*left*), corresponding phase images (*middle*), and |*F*| (*right*) of non-metastatic primary prostate epithelial cells (PrEC) and highly metastatic prostate cancer cells (PC3). (C) Representative traction maps (*left*), corresponding phase images (*middle*), and |*F*| (*right*) of non-metastatic bronchial epithelial cells (BEAS2B) and metastatic lung adenocarcinoma cells (A549). All cells are on polyacrylamide substrates with Young's Modulus (E) = 5 kPa and type I collagen concentration of 0.1 mg/mL. Scale bar = 50 µm. Mean+SEM; *** indicates p<0.001.

At a moderate stiffness mimicking tumorigenic conditions in breast tissue (E = 5 kPa) [15], measurements of cellular force using TFM indicate that MDAMB231 highly metastatic breast cancer cells exhibited stronger traction stresses compared to the non-metastatic MCF10A mammary epithelial cells (Fig. 1A). Likewise, both the PC-3 highly metastatic prostate cancer cells (Fig. 1B) and the A549 metastatic lung cancer cells (Fig. 1C) exhibited significantly greater traction stresses than the non-metastatic PrEC primary prostate epithelial cells and the BEAS-2B lung epithelial cells, respectively. These data suggest that increasing force generation in cells of high metastatic potential may be a biophysical characteristic of metastatic cells that could potentially act as a mechanical marker for metastasis.

### Substrate stiffness and collagen density mediate magnitude of traction force

**Figure 2:**
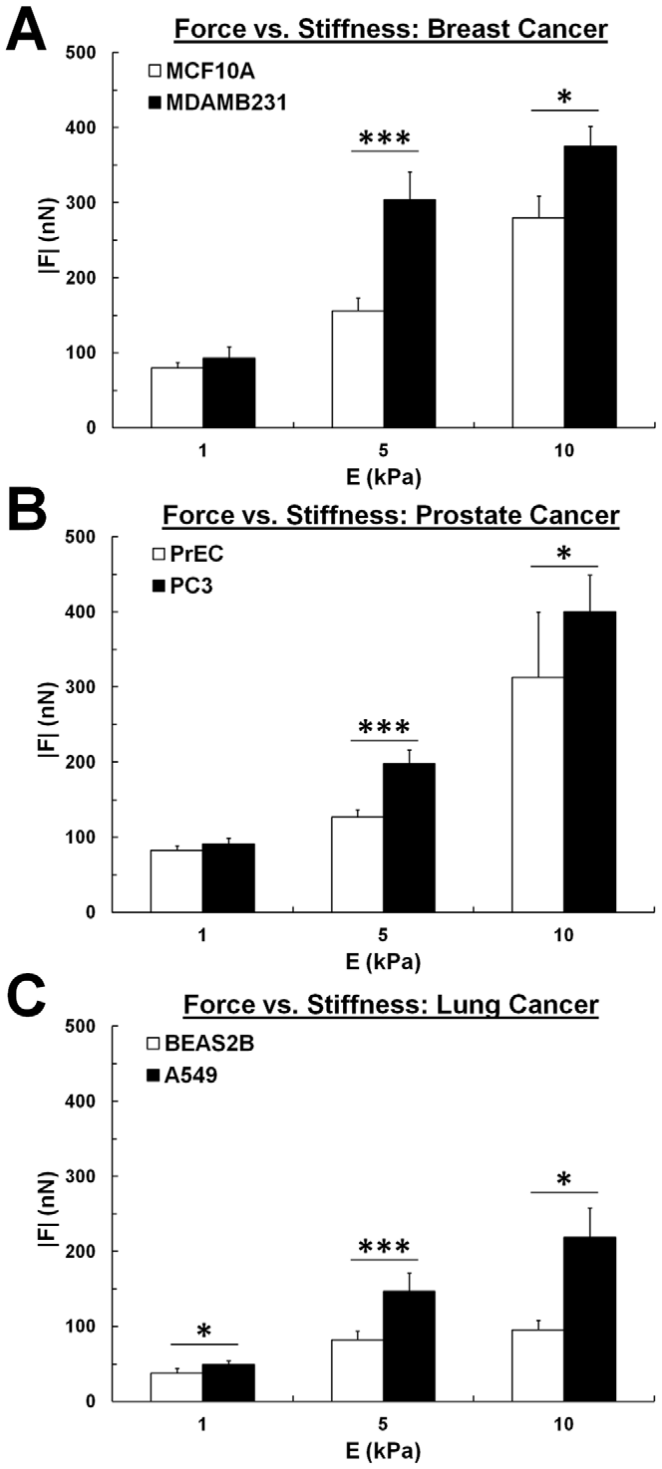
Increased matrix stiffness contributes to increased force generation. Net traction force (|*F*|) increases with increasing substrate stiffness (E = 1–10 kPa) for (A) MCF10A non-metastatic mammary epithelial cells and MDAMB231 metastatic cancer cells, (B) PrEC non-metastatic primary prostate epithelial cells and PC3 metastatic prostate cancer cells, and (C) BEAS2B non-metastatic bronchial epithelial cells and A549 metastatic lung cancer cells. Ligand density is maintained at 0.1 mg/mL collagen I. 5 kPa data is from Figure 1. Note also that the metastatic cancer cells (black) exert greater forces than non-metastatic cells (white) at all matrix stiffness levels studied. Mean+SEM; * indicates p<0.05; *** indicates p<0.001.

Because cancer cells encounter heterogeneous environments exhibiting a range of stiffness during metastatic progression *in vivo*, such as stiff, matrix-dense regions near the tumor and compliant adipose tissue during stromal invasion [15], we measured the traction forces exerted on substrates of varying stiffness (E = 1–10 kPa), with collagen density being held constant (0.1 mg/mL). We found that, in addition to exerting greater forces on substrates of tumorigenic stiffness (Fig. 1A), MDAMB231 cells also have a greater overall net traction force (|*F*|) than MCF10A cells when cultured on substrates of higher stiffness (E = 10 kPa, Fig. 2A). This trend was also recapitulated with the prostate and lung cancer models, in which the metastatic PC3 (Fig. 2B) and A549 (Fig. 2C) prostate and lung cancer cells exerted significantly greater forces than non-metastatic PrEC and BEAS2B cells on stiff substrates. On more compliant substrates (E = 1 kPa), the metastatic cells of each cancer type studied exert slightly higher forces than their non-metastatic counterparts. Additionally, we observed a significant increase in net traction force with increasing substrate stiffness within all 6 cell lines (p<0.01), suggesting that the stiffness of the environment surrounding cancer cells can directly contribute to the amount of traction force exerted at a single cell level. These data indicate that the stiffness of the microenvironment significantly affects the magnitude of forces generated by metastatic and non-metastatic cells, and suggest that, in general, metastatic cells are able to exert greater net traction forces than non-metastatic cells.

**Figure 3:**
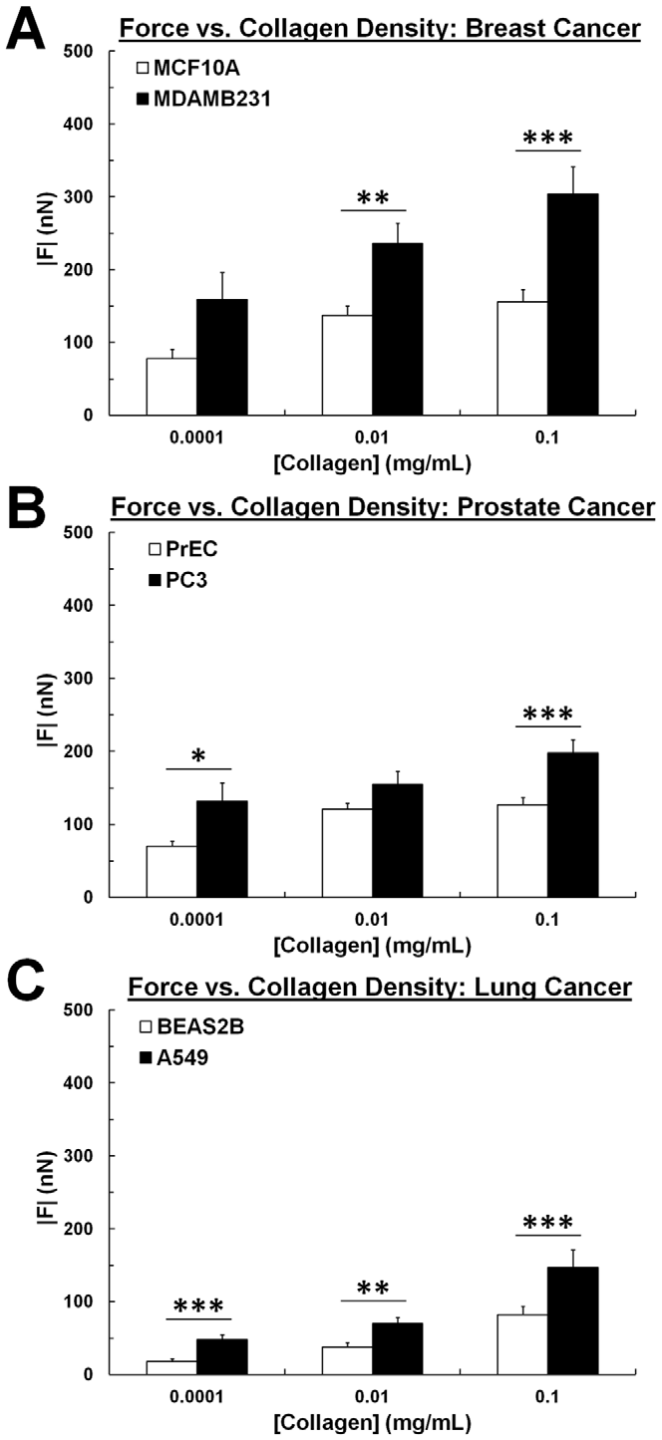
Increased collagen density contributes to increased force generation. Net traction force (|*F*|) increases with collagen density (0.0001–0.1 mg/mL collagen I) for (A) MCF10A non-metastatic mammary epithelial cells and MDAMB231 metastatic cancer cells, (B) PrEC non-metastatic primary prostate epithelial cells and PC3 metastatic prostate cancer cells, and (C) BEAS2B non-metastatic bronchial epithelial cells and A549 metastatic lung cancer cells. Stiffness is maintained at E = 5 kPa. 0.1 mg/mL collagen I data is from Figure 1. Note also that the metastatic cancer cells (black) exert greater forces than non-metastatic cells (white) at all collagen densities studied. Mean+SEM; * indicates p<0.05; ** indicates p<0.01; *** indicates p<0.001.

Collagen content of the microenvironment also changes throughout neoplastic progression [13], with increased collagen expression particularly noted in clinical analyses of high grade tumors [10]. As such, we examined the effect of collagen density on cancer cell traction forces. Using substrates of constant stiffness (E = 5 kPa) and varying collagen density (0.0001–0.1 mg/mL), we found that metastatic MDAMB231 breast cancer cells exert a greater net traction force (|*F*|) than non-metastatic MCF10A cells across the entire range of collagen densities tested (Fig. 3A). This trend is recapitulated with both the prostate and the lung cells, with metastatic PC3 (Fig. 3B) and A549 (Fig. 3C) prostate and lung cancer cells exerting greater forces than their non-metastatic counterparts, PrEC and BEAS2B cells, on substrates with varying collagen density. Similar to the previously observed trend of increasing force with increasing stiffness, all 6 cell lines also showed a significant increase (p<0.01) in net traction force with increasing collagen density, suggesting that increased collagen content within the tumor microenvironment can also drive force generation of cancer cells. Together these data indicate that metastatic and non-metastatic cells have differential force profiles that are significantly affected by mechanical and chemical matrix properties of the tumor microenvironment.

### Differences in force generation are not correlated to differences in cell spreading

**Figure 4:**
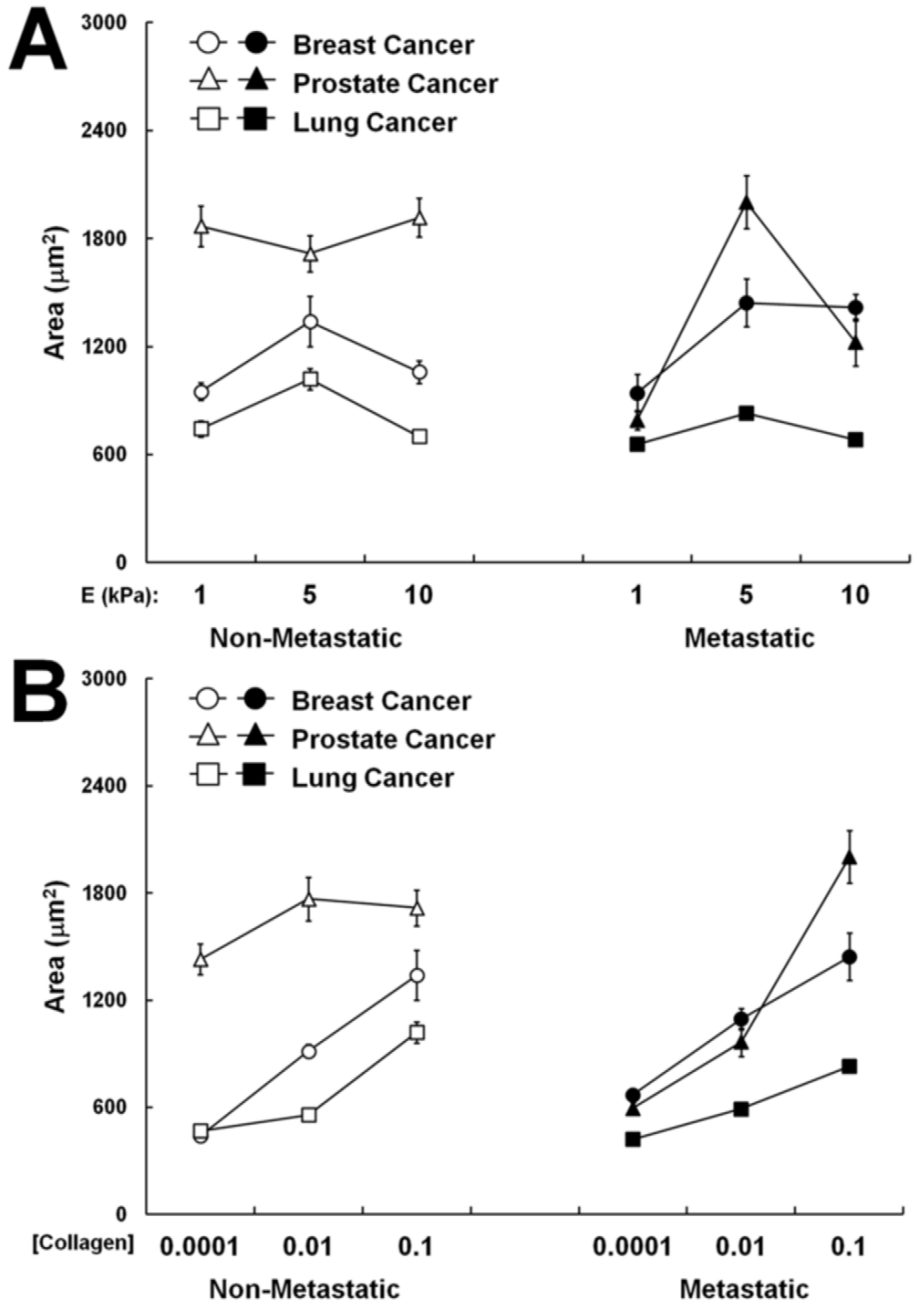
Cell area is differentially altered by matrix stiffness and collagen density. Projected cell area shows a biphasic relationship with substrate stiffness (A) and a direct relationship with collagen density (B) in the majority of the metastatic (black) and non-metastatic cells (white) studied. No consistent trend was observed when comparing the projected cell area of complementary metastatic and non-metastatic cells.

Because cellular force generation has been linked to cell spread area in previous studies [34], [36], we assessed whether increased cellular traction force in metastatic cells on both stiff matrices and those displaying high densities of collagen was simply caused by an increase in cell area (i.e. that larger cells exert higher forces). The area of isolated cells was measured during the TFM experiments previously described. We observed no significant or consistent trend when comparing the spreading area of metastatic breast, prostate, and lung cancer cells to their non-metastatic epithelial cell counterparts across substrates of constant collagen density and varying stiffness (Fig. 4A) or across substrates of constant stiffness and varying collagen density (Fig. 4B) (p>0.05). These data indicate that the observed increases in traction force in metastatic versus non-metastatic cells are not linked to cell area. Interestingly, we noted that five of the six cell lines (excluding the non-metastatic PrEC prostate epithelial cells) exhibited a biphasic relationship with substrate stiffness, with the maximum cell spreading area occurring on polyacrylamide substrates of an intermediate stiffness (E = 5 kPa) (Fig. 4A). In contrast, we observed that these same five cell lines exhibited a direct relationship between spreading area and collagen density across the range of 0.0001–0.1 mg/mL, with increased area correlating with increased collagen density (Fig. 4B).

**Figure 5:**
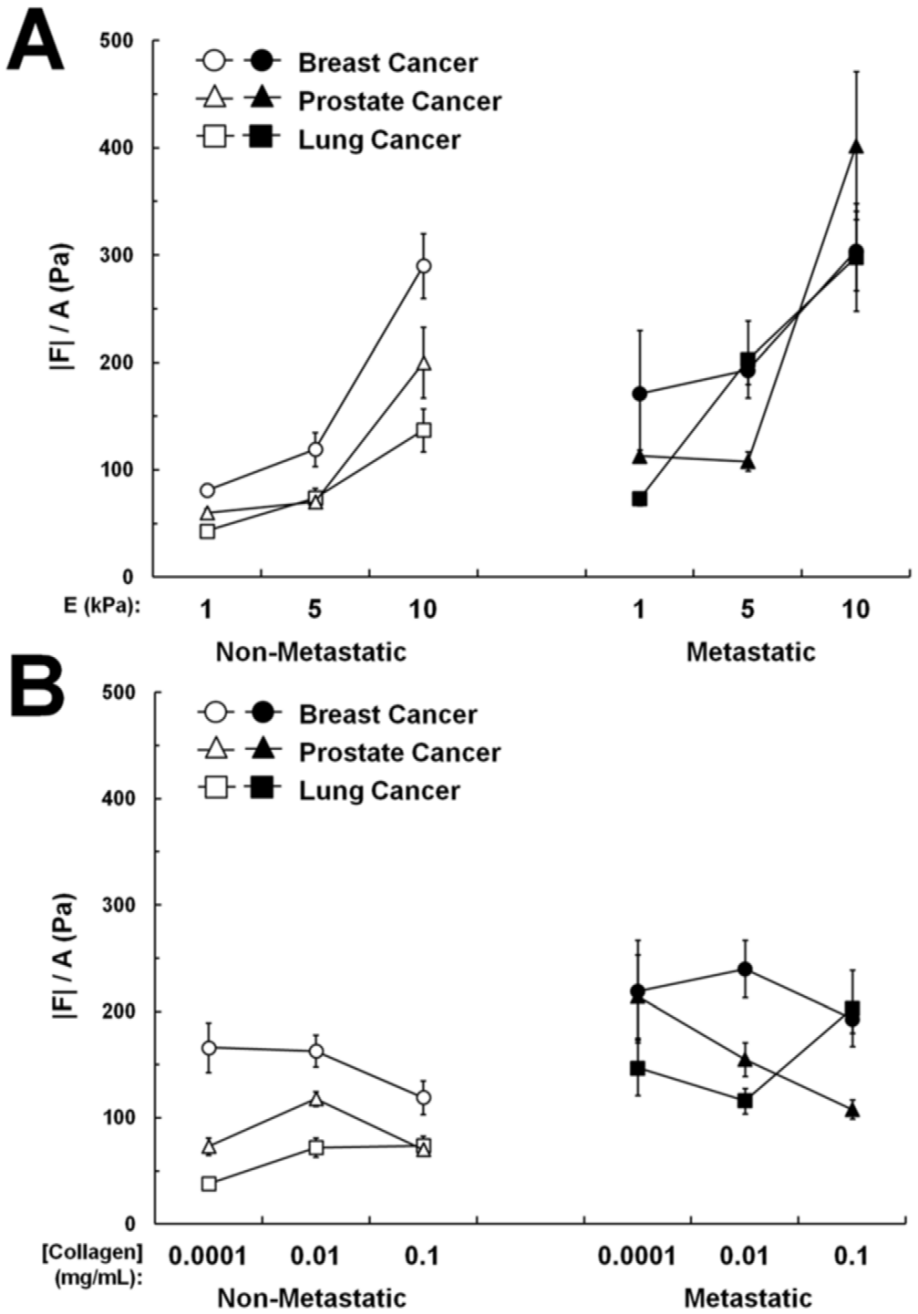
Average traction stress increases with stiffness, not collagen density. |*F*| of each cell is normalized by its projected area (|*F*|/A) as a measurement of average traction stress. Average traction stresses increase with increasing substrate stiffness (A) but become relatively uniform with increasing collagen density (B) in both metastatic (black) and non-metastatic (white) cells studied.

To further investigate the role of cell area in the generation of traction forces, we analyzed the net traction force (|*F*|) of each cell normalized by its projected area (|*F*|/A) as a measurement of average traction stress. When the traction forces of cells plated on substrates of variable stiffness were normalized by their respective areas, average traction stress increased with increasing stiffness (p<0.0001, Fig. 5A). Therefore, the increased force on stiffer substrates is not due to increased cell spreading. However, the average traction stress of cells plated on substrates of variable collagen density revealed an overall equalization of forces across the collagen concentrations tested (Fig. 5B). These data suggest that increased collagen density may promote force generation by causing an increase in cell spreading.

### Metastatic cells exert increased force in an isogenic model

**Figure 6:**
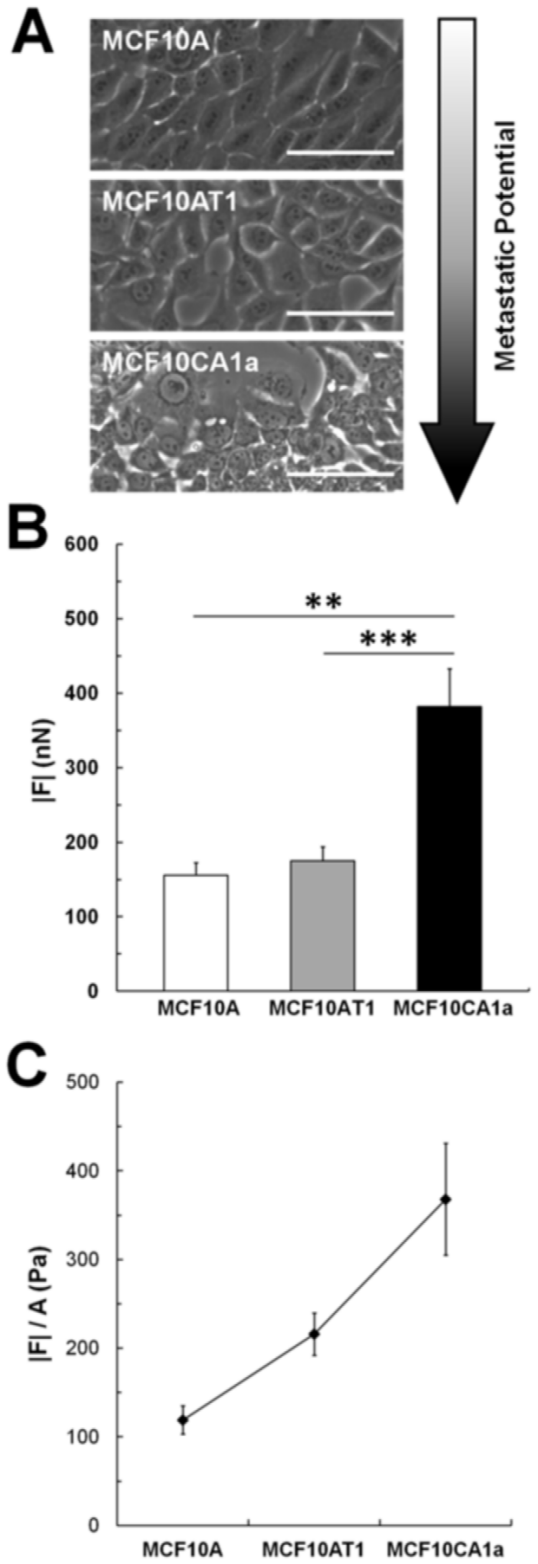
Metastatic derivative in a series of isogenic cell lines exerts greater forces. (A) Phase images of parental untransformed cells (MCF10A), transformed premalignant (MCF10AT1) and highly metastatic (MCF10CA1a) derivatives. (B) Net traction forces increase with increasing metastatic potential, with the highest forces exerted by the metastatic MCF10CA1a cells. (C) Average traction stress (|*F*|/A) increases with increasing metastatic potential. Mean ± SEM; ** indicates p<0.01; *** indicates p<0.001.

Because the cell lines used in these experiments are of genetically distinct lineages, we further investigated our hypothesis that metastatic cells generated increased traction forces compared to non-metastatic cells using an isogenic cell model: the 10AT series of cell lines. The 10AT series consists of several well-characterized cell lines which represent the full spectrum of neoplastic progression [39]. We focused on the MCF10AT1 and MCF10CA1a lines [39], [44], [45], compared to their parental spontaneously immortalized MCF10A cells used previously in this work. MCF10AT1 cells were derived from MCF10A cells transfected with the constitutively active Ha-ras oncogene. These cells are considered ‘premalignant’ and will first form simple ducts in nude mice xenografts, followed by benign lesions which occasionally progress to carcinomas [44]. MCF10CA1a cells were selectively derived from the MCF10AT1 carcinoma populations. These cells form undifferentiated carcinomas and rapidly growing metastases within the lungs [39], [44], [45]. Together, the MCF10A, MCF10AT1, and MCF10CA1a are isogenic human cell lines representative of different stages of metastatic potential: non-tumorigenic, premalignant, and highly metastatic (Fig. 6A). The highly metastatic MCF10CA1a derivative exerted significantly greater traction forces compared to the benign MCF10AT1 and non-tumorigenic parental MCF10A cells (Fig. 6B) on substrates mimicking the stiffness of tumorigenic breast tissue (E = 5 kPa) [15]. When the net traction force was normalized by projected area, there was a significant increasing trend in average traction stress (p<0.001, Fig. 6D). Overall, these data from an isogenic cell model correlate well with the data from breast, prostate, and lung cells derived from different genetic backgrounds (Fig. 1).

## Discussion

To date, there is no universal molecular marker of metastasis. Because of the known role of cellular force in cell migration, we explored traction force generation as a potential biophysical marker characteristic of cells with metastatic ability. The results presented here show that highly invasive breast, prostate, and lung cancer cells exert significantly greater traction forces than their non-invasive epithelial cell counterparts on 2D substrates of physiologically relevant stiffness and varying collagen density. These findings indicate that inherent force differences exist in cells of differing metastatic potential. Additionally, increased matrix stiffness and ligand density promote increased contractile force generation in contrasting ways: collagen density appears to lead to increased cellular force generation by directly mediating cell spreading, while matrix stiffness appears to increase cell forces independently of cell spreading.

Previous studies by others have also explored various biophysical traits as potential markers of metastasis. Atomic force microscopy (AFM) and optical stretching measurements have shown that metastatic cancer cells are more compliant than benign cells, both in established cell lines [46], [47] and in primary tumor samples [48]. These data allow speculation that a difference in the plasticity of cancer cells may contribute to their enhanced migration away from the primary tumor by allowing them to more easily maneuver through the ECM. These studies are the first indications that there are mechanical properties inherent in some cancer cells that may promote malignant and metastatic behavior. In our study, we explore another aspect of cell mechanics, cellular force generation, to show that metastatic cancer cells from a variety of cancer types exert greater traction stresses than their non-metastatic counterparts. We speculate that these higher forces could potentially drive cancer progression by promoting cellular behaviors such as cell migration, adhesion, and ECM remodeling during metastatic invasion.

Cellular mechanical properties can also be affected by the microenvironment. Using particle-tracking microrheology, Baker *et al.* showed that cells embedded in 3D matrices are stiffer than those plated on 2D substrates [29]. They also show that 3D matrix stiffness and collagen density significantly affect the intracellular tension in tumor cells, although this relationship is dependent on both the type of cell and the cell's transformation state [29], [49]. Increased matrix stiffness in turn has been shown to enhance cell motility in 2D and 3D [28], [30], while decreasing the stiffness of the tumor microenvironment has been shown to inhibit malignant progression [13], [15]. Increased ECM density has been identified as a risk factor for breast cancer [37], and has also been shown to promote invasion by enhancing integrin clustering, upregulating Rho and PI3 kinase activity, and increasing focal adhesion formation [13], [15]. Recently, Levental et. al. has shown that inhibiting collagen crosslinking increases tumor latency, reduces cell proliferation, and reduces tumor volume and incidence in a mouse model [13]. In this work, we have shown that matrix stiffness and collagen density both mediate cellular force generation, with cells exerting greater force on substrates with either increased stiffness or increased collagen density.

Since both matrix stiffness and ligand density have been associated with increased cell spreading [34], [35], [36], [50], [51], we examined their effects on cell spreading area here. We find that in the majority of the cell lines employed in this study, there existed a biphasic relationship between cell spreading and substrate stiffness (Fig. 4A), but a direct relationship between cell spreading and collagen density (Fig. 4B). While the relationship between area and collagen density is consistent with previous studies in numerous cell types [34], [35], [36], the relationship between substrate stiffness and cell spreading contrasts with similar studies performed on several different non-cancerous cell types [34], [50], [51]. However, there has been some evidence reported previously that the effect of matrix stiffness on cell spreading is highly variable and cell type- specific, particularly in cancer cells [52].

Interestingly, we observe that cells on substrates of increasing stiffness exhibit increased average traction stresses, while cells on substrates of increasing collagen density exhibit no change in average traction stresses (Fig. 5). We speculate that matrix stiffness is able to promote force generation through a mechanosensing-mechanism which causes an upregulation of contractility-related proteins, while collagen density promotes force generation by controlling the number of available integrin binding sites available to the cell, therefore directly mediating the cell spreading area and allowing larger cells to exert greater forces. Importantly, metastatic cancer cells exert increased force compared to non-metastatic cells regardless of cell spreading area. This suggests increased force generation is inherently tied to the metastatic phenotype regardless of the microenvironment. It is also important to note that while this data is acquired in a 2D environment, previous work in our lab has shown that forces in 2D reflect the forces generated within a 3D environment [42].

While we chose to focus on three of the most common metastatic cancers (breast, prostate, and lung), we cannot claim that contractile force will be a universal predictor of metastatic behavior without thoroughly characterizing every type of cancer. Indeed, recent work has shown that the method of transformation or exposure to different tumorigenic signals can significantly affect cellular mechanical properties in contrasting ways [49], [53]. Additionally, a recent study using a series of murine breast cancer cell lines of increasing metastatic potential observed the reverse trend to our data, showing that cells of increasing metastatic potential exert weaker contractile forces [54]. While it is an elegant model of metastatic potential, the relevance of mouse-derived cancers to cancers which develop within the human body continues to be disputed [55]. Based on the data shown in this study, particularly on the work done using the isogenic MCF10AT series of cell lines, we believe that pursuing traction force generation as a mechanical indicator of metastasis could potentially reveal mechanistic details that will lead to a better understanding of the initiation and progression of metastatic cancer. Additionally, examining proteins which are known to play key roles in mediating cell force, such as RhoA, ROCK, or myosin light chain phosphatase, may reveal a protein marker for traction stresses which could be more directly employed clinically to diagnose metastasis. Further studies will be needed on a broader range of cancer cells to determine the applicability of this kind of marker to a wide variety of cancers.

In conclusion, we have shown that metastatic cells exert significantly greater traction forces than non-metastatic cells in breast, prostate, and lung cancer models, and that these forces can be driven by the mechanical and chemical properties of the tumor microenvironment. These findings suggest that inherent force differences exist in cells of differing metastatic potential, and that these differences could be developed into a biophysical marker that could be used to determine the likelihood of metastasis. Additionally, our data suggest that identifying a mechanism to therapeutically target cellular force may be a promising avenue for inhibiting metastatic progression.
